# Supplementary material for: Development and validation of the Health Promoting Behaviour for Bloating (HPB-Bloat) scale
Source: PeerJ. 2021 Jun 4;9:e11444. doi: 10.7717/peerj.11444 (PMC8183425; doi:10.7717/peerj.11444)
Supplement: Supplemental Information 3 [file peerj-09-11444-s003.pdf]

Perlakuan peningkatan kesihatan dalam meredakan kembung perut.
"Health promoting behaviour for abdominal bloating (HPB-Bloat)"

Arahan: Borang ini mengandungi 17 soalan untuk meneroka apa tindakan yang dipercayai membantu untuk menyembuhkan kembung perut. Sila kadarkan diri anda berdasarkan apa yang pernah anda lakukan sebelum ini dan, jawapan mungkin berbeza antara individu. Sila berkongsi apa yang membantu anda menyembuhkan kembung perut. Terdapat lima jawapan yang berkemungkinan untuk dipilih:

"Instruction: This form consists of 17 questions to explore the actions which believed to help reduce abdominal bloating. Please rate yourself based on what you have done previously and, the answers may vary by individuals. Please shared what helps you to improve abdominal bloating. There are five answers which could be selected:"

	Tidak pernah *" Never"*	Jarang-jarang *" Seldom"*	Kadang-kadang *" Sometimes"*	Kerap *" Often"*	Sangat Kerap *" Very often"*
1.Saya makan buah-buahan atau sayur-sayuran yang boleh mengurangkan kembung perut secara semulajadi (timun, tembikai, atau seumpamanya).<br>"*I eat fruits and vegetables that can help improve abdominal bloating naturally (cucumber, watermelon and etc)."*	1	2	3	4	5
2.Saya memberitahu sebarang tanda luar biasa yang berkait dengan perut kepada doktor.<br>"*I told any unusual sign that related to stomach to a doctor."*	1	2	3	4	5
3.Saya mengambil bahagian dalam aktiviti fizikal rekreasi (seperti berbasikal, bermain permainan tradisional, seni mempertahankan diri, tarian atau seumpamanya).<br>"*I took part in recreational physical activity (like cycling, playing tradisional game, martial arts, dance or etc)."*	1	2	3	4	5
4. Saya keluar berjalan apabila saya tertekan.<br>*"I go for a walk when I feel stressed."*	1	2	3	4	5
5.Saya akan sentiasa bersedia untuk mencuba teknik-teknik rawatan baharu untuk mengurangkan masalah kembung perut apabila perlu.<br>"*I will be read to try new technique's to reduce bloating problems when needed."*	1	2	3	4	5
6.Saya makan makanan probiotik 'bakteria baik" seperti yogurt, yang menyelesaikan masalah penghadaman.<br>*" I eat "good bacteria" probiotic food like yogurt, that help solve digestive problem."*	1	2	3	4	5
7.Saya mengikut nasihat doktor berkait penjagaan kesihatan.<br>*"I follow doctor's advice related to health management."*	1	2	3	4	5
8.Saya melakukan senaman ringan sekurang-kurangnya 3 kali seminggu.<br>"*I did light warm up at least three times per week."*	1	2	3	4	5
9.Saya belajar bagaimana menguruskan tekanan daripada buku, atau bahan ilmiah (majalah kesihatan, rancangan kesihatan, tv).<br>*"I learnt how to manage stress from books, or educational books (health magazines, health tv program, tv)."*	1	2	3	4	5
10.Saya makan makanan yang tidak menyebabkan kembung perut seperti	1	2	3	4	5

halia, almond, oren, pisang, timun susu, teh herba atau seumpamanya).<br>*"I eat food that didn't cause bloating like ginger, almond, orange, banana, honey dew, herbs tea or etc."*					
11.Saya berbincang tentang kesihatan saya dengan doktor.<br>*"I discussed about my health with a doctor."*	1	2	3	4	5
12.Saya bersenam sambil melakukan aktiviti harian (berjalan semasa waktu makan tengahari, menggunakan tangga selain lif, meletakkan kenderaan jauh dari destinasi dan berjalan atau seumpamanya).<br>*"I exercised while doing daily life routine (walking during lunch, using stairs instead of lift, park cars far from the destination and walk or etc)."*	1	2	3	4	5
13.Saya mendengar muzik untuk mengurangkan tekanan.<br>*"I listened to music to reduce stress."*	1	2	3	4	5
14.Saya mengamalkan pengambilan serat dalam pemakanan saya (sayur hijau, atau seumpamanya).<br>*"I regularly take fiber in meals (green vegetable, or etc)."*	1	2	3	4	5
15.Saya meminta maklumat yang lebih daripada doktor mengenai cara menyembuhkan kembung perut.<br>*"I asked for additional information from a doctor about ways to heal abdominal bloating."*	1	2	3	4	5
16.Saya mengelak dari makan makanan yang menyebabkan kembung perut/gas (seperti minuman berkarbonat, gula-gula getah, pemanis tiruan atau seumpamanya).<br>*"I avoid from eating food that can caused bloating/gas (like carbonated drink, bubble gun, sweetener or etc)."*	1	2	3	4	5
17.Saya rehat sekejap atau tidur apabila tertekan.<br>"*I rest a bit or sleep when stressed."*	1	2	3	4	5

Note:
Factors and items
1) Diet: 1, 6, 10, 14, 16
2) Health and treatment awareness: 2, 5, 7, 11, 15
3) Physical activity: 3, 8, 12
4) Stress management: 4, 9, 13, 17
